# Tribute to a True Mentor: William Maixner *Our Life and Times…* A Tribute in Memory of William Maixner (1952–2020), a Man of Many Talents

**DOI:** 10.3389/fpain.2021.808014

**Published:** 2021-12-13

**Authors:** Denniz Asad Zolnoun

**Affiliations:** Department of Plastic and Reconstructive Surgery, The University of North Carolina at Chapel Hill, Chapel Hill, NC, United States

**Keywords:** mentor, memorandum, tribute, eulogy, pain mechanism researcher, pioneer

Bill Maixner, DDS, PhD, the Joannes H. Karis, MD, Professor of Anesthesiology at Duke, was a world-renowned pioneer in pain research.

Much has been written of Bill's contributions in basic and clinical research [over 300 published papers: (1)], his extensive extramural support, including numerous NIH grants reflecting continuous funding since 1986, notable awards such as the NIH National Center of Excellence program project grants (PPG), and his accolades throughout his career, including the New York College of Dentistry Distinguished Scientist Award and the Wilbert E. Fordyce Clinical Investigator Award from the American Pain Society. His background in dentistry made his work in the area of temporomandibular joint syndrome an obvious focus of his research interests. He was principal investigator on two National Institute of Dental and Craniofacial Research (NIDCR) studies of pain in TMJ disorders. However, what made Bill truly remarkable was his transcendence of the orofacial realm of dentistry and his impact on generations of collaborators and disciples.

Bill was a pioneer in conceptualizing pain as a systemic disorder, rather than focusing exclusively on a single part of the body. He assembled a diverse team of researchers and clinicians to identify commonalities and unique aspects of pain across diverse populations of pain patients, co-authoring papers on knee pain, arthritis, migraines, fibromyalgia, and other pain syndromes, with particular attention to comorbidities among disorders. He used this broad-minded approach to study genetic signatures and physiological pathways associated with pain, in search of improved diagnosis and treatment.

Bill served on the Foundation for Anesthesia Education and Research's (FAER) Academy of Research Mentors in Anesthesiology, reflecting his dedication to career guidance and mentorship. Bill invested his most precious commodity, time, in the dozens—perhaps hundreds—of fellows and students across multiple disciplines to whom he provided direction and support in the paths they were taking in their own lives, both professionally and personally.

I was one of those beneficiaries. More than two decades ago, I approached him with a crazy idea that gynecological pain might be linked to orofacial pain. As a clinical fellow with no research pedigree, I had noticed that a sizable proportion of patients with pelvic pain also suffered from pain in the orofacial region, and I wondered whether the overarching mechanism of pain with jaw movement might also cause pain during intercourse. Bill gave me his undivided attention. He asked about the parallels I saw not only in the patients' pain, but also in the historical treatment for pain of unknown origin—remove the painful tissue and see if that fixes the problem.

Bill welcomed me as a member of his core research team. It might have looked a bit odd to have a gynecologist on a dental research team, but in his calm and understated style he never wavered on his commitment toward me as a mentor. He took every opportunity to facilitate my scholarly interaction with other giants in the field. He created a community of clinicians across many disciplines—anesthesia, rheumatology, functional GI, alternative medicine, chiropractic, psychology, veterinary medicine, autism—where we learned from one another and focused on the similarities in our respective silos. Slowly but surely, we all began to understand his vision for individualized medicine for patients suffering from myriad of chronic pain conditions.

Bill was also kind and generous. He opened his home on holidays, and we celebrated and laughed together. I was one of many direct recipients of his and his family's hospitality over the years.

When I first discussed my patients' pain during intercourse, Bill had blushed, so I developed the euphemism “voluntary movement of pelvic muscles.” This did not prevent him from blushing. But Bill was a champion for women with pelvic pain, recognizing the importance of identifying specific mechanisms to explain suffering that was often dismissed in medicine. He will be sorely missed.

I will end with excerpts from a letter I wrote to Bill after his death, to tell him what he meant to me as a person:

Dr. Maixner, I miss hearing your voice and your chuckle. I miss seeing you blush during our mentoring session when I asked for your guidance on neurosensory assessment experiments in the urogenital region. Because of you, I have been able to draw parallels between the biology, pathology, and functional anatomy of the “nether regions” and of other parts of the body. You were always steps ahead, anticipating my educational and career development needs.

I never got a chance to say goodbye and tell you what you meant to me. But I want you to know that I and others will honor your legacy by continuing your life's work on mechanism-based classification of pain based on individual patients' clinical and genotypic profile. I promise you I(we) will get those papers out!

## Author Contributions

The author confirms being the sole contributor of this work and has approved it for publication.

## Conflict of Interest

The author declares that the research was conducted in the absence of any commercial or financial relationships that could be construed as a potential conflict of interest.

## Publisher's Note

All claims expressed in this article are solely those of the authors and do not necessarily represent those of their affiliated organizations, or those of the publisher, the editors and the reviewers. Any product that may be evaluated in this article, or claim that may be made by its manufacturer, is not guaranteed or endorsed by the publisher.

## References

[1] Ji RR Nackley A Huh Y Terrando N Maixner W. Neuroinflammation and central sensitization in chronic and widespread pain. Anesthesiology. (2018) 129:343–66. 10.1097/ALN.0000000000002130 29462012 PMC6051899

[2] Duke University School of Medicine. Duke Anesthesiology (2021). Available online at: https://anesthesiology.duke.edu/?p=855809 (accessed November 2, 2021).

